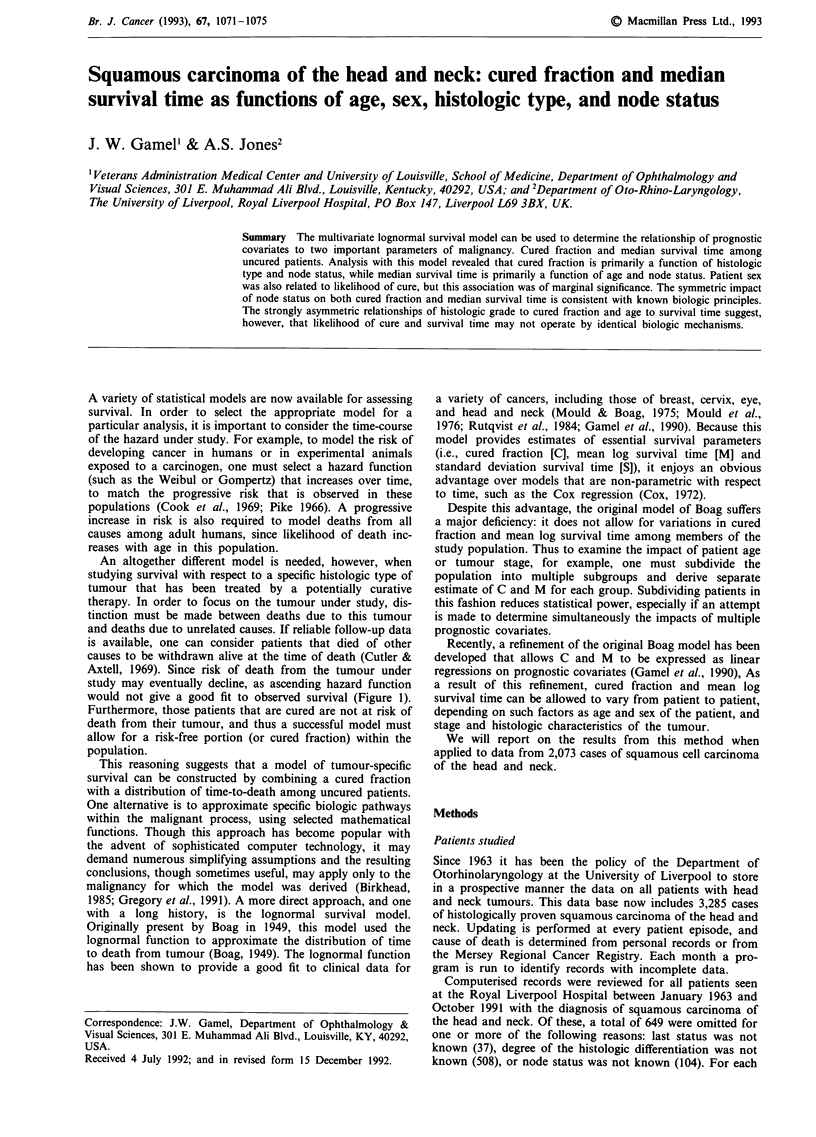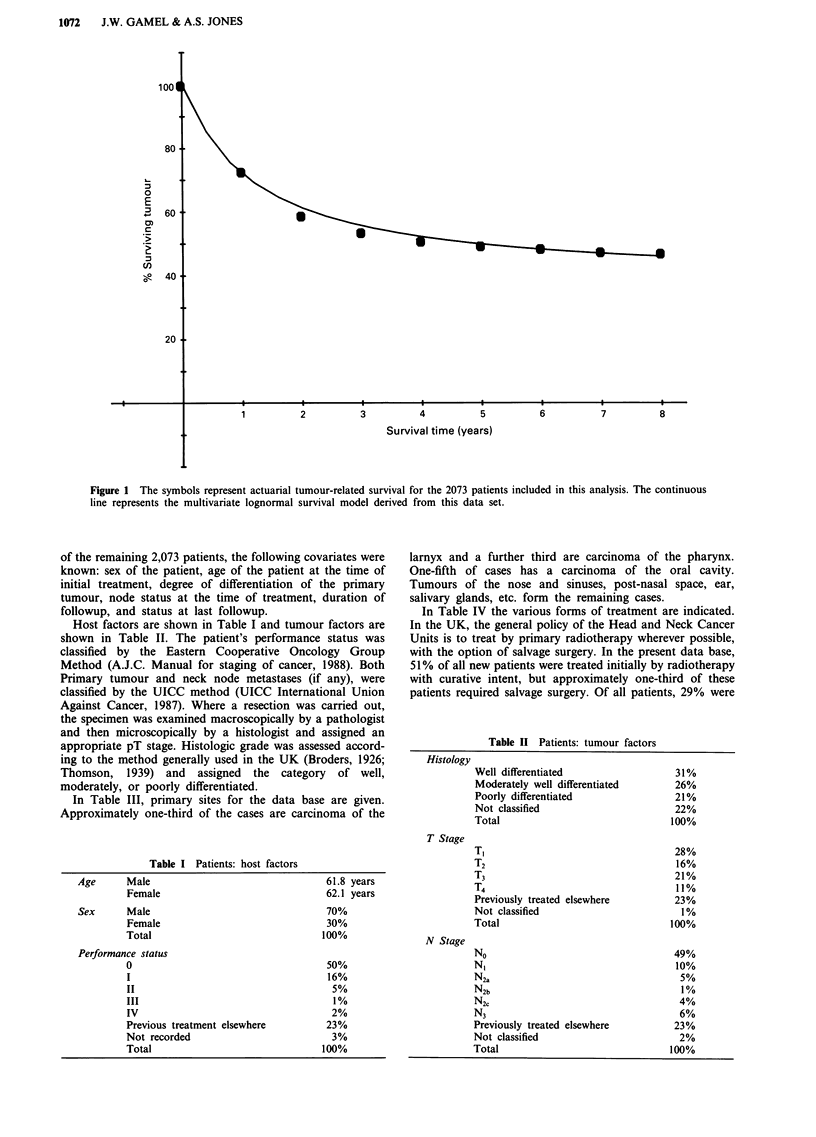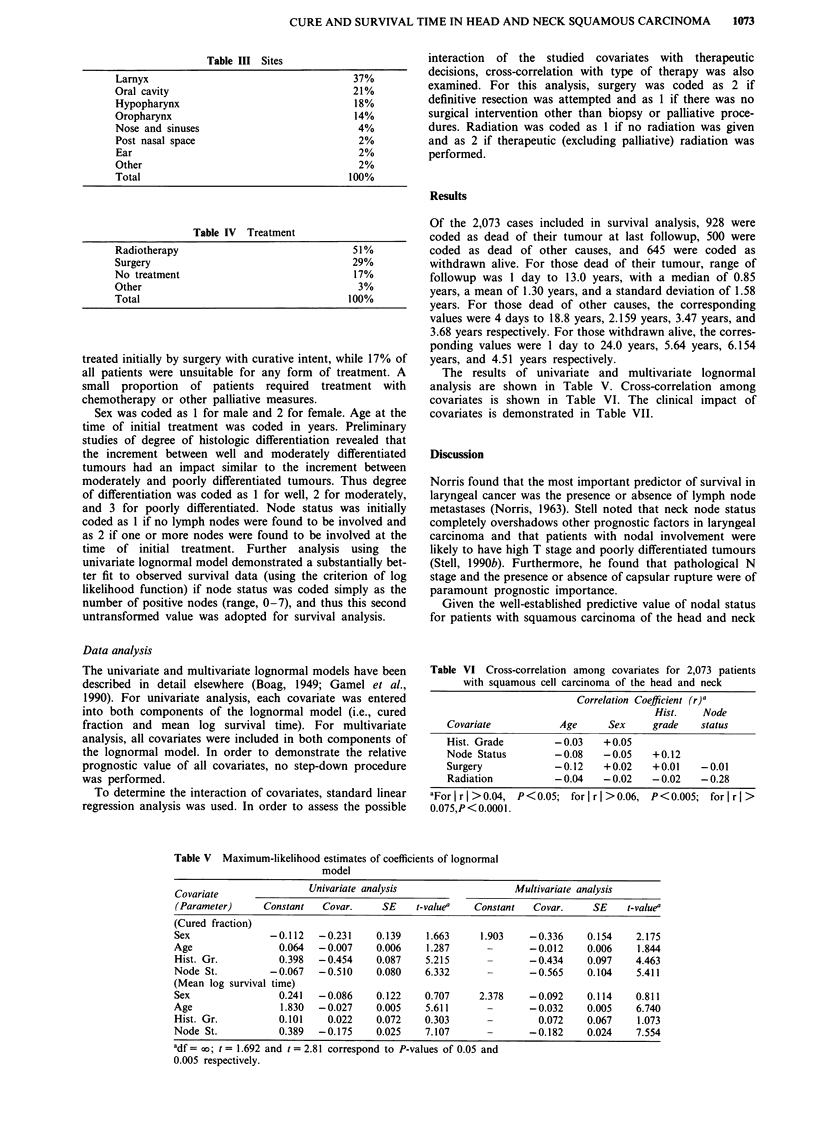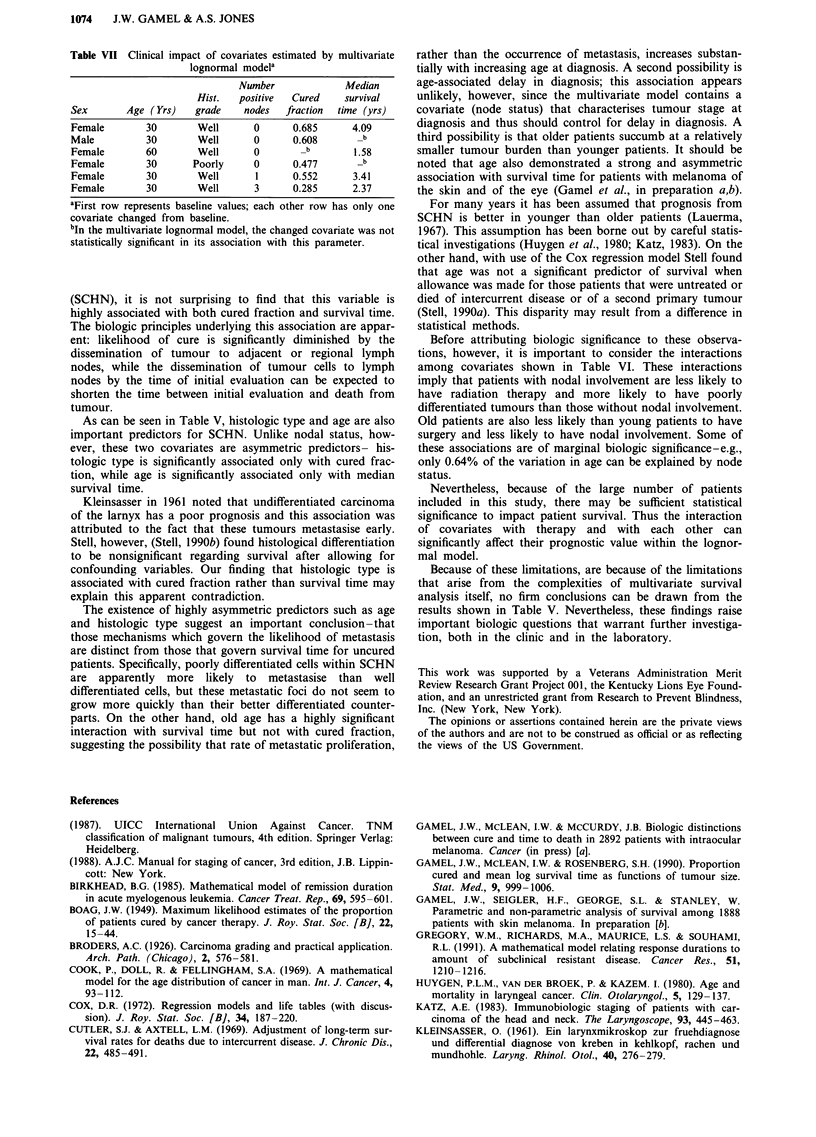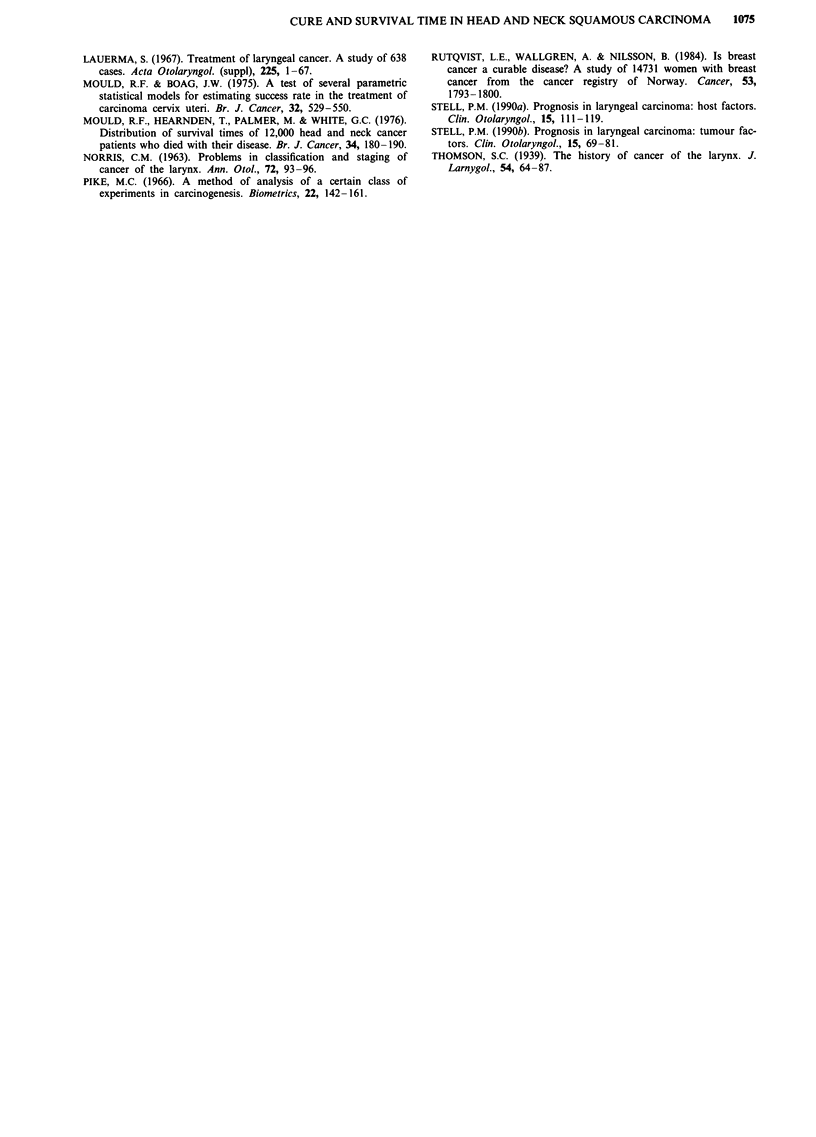# Squamous carcinoma of the head and neck: cured fraction and median survival time as functions of age, sex, histologic type, and node status.

**DOI:** 10.1038/bjc.1993.196

**Published:** 1993-05

**Authors:** J. W. Gamel, A. S. Jones

**Affiliations:** Veterans Administration Medical Center, Louisville, Kentucky.

## Abstract

The multivariate lognormal survival model can be used to determine the relationship of prognostic covariates to two important parameters of malignancy. Cured fraction and median survival time among uncured patients. Analysis with this model revealed that cured fraction is primarily a function of histologic type and node status, while median survival time is primarily a function of age and node status. Patient sex was also related to likelihood of cure, but this association was of marginal significance. The symmetric impact of node status on both cured fraction and median survival time is consistent with known biologic principles. The strongly asymmetric relationships of histologic grade to cured fraction and age to survival time suggest, however, that likelihood of cure and survival time may not operate by identical biologic mechanisms.


Br. J. Cancer (1993), 67, 1071-1075 © Macmillan Press Ltd., 1993

### Squamous carcinoma of the head and neck: cured fraction and median survival time as functions of age, sex, histologic type, and node status

J. W. Gamel[1] & A.S. Jones[2]

[1]*Veterans Administration Medical Center and University of Louisville, School of Medicine, Department of Ophthalmology and Visual Sciences, 301 E. Muhammad Ali Blvd., Louisville, Kentucky, 40292, USA; and* [2]*Department of Oto-Rhino-Laryngology, The University of Liverpool, Royal Liverpool Hospital, PO Box 147, Liverpool L69 3BX, UK.*

**Summary** The multivariate lognormal survival model can be used to determine the relationship of prognostic covariates to two important parameters of malignancy. Cured fraction and median survival time among uncured patients. Analysis with this model revealed that cured fraction is primarily a function of histologic type and node status, while median survival time is primarily a function of age and node status. Patient sex was also related to likelihood of cure, but this association was of marginal significance. The symmetric impact of node status on both cured fraction and median survival time is consistent with known biologic principles. The strongly asymmetric relationships of histologic grade to cured fraction and age to survival time suggest, however, that likelihood of cure and survival time may not operate by identical biologic mechanisms.

A variety of statistical models are now available for assessing survival. In order to select the appropriate model for a particular analysis, it is important to consider the time-course of the hazard under study. For example, to model the risk of developing cancer in humans or in experimental animals exposed to a carcinogen, one must select a hazard function (such as the Weibul or Gompertz) that increases over time, to match the progressive risk that is observed in these populations (Cook *et al.*, 1969; Pike 1966). A progressive increase in risk is also required to model deaths from all causes among adult humans, since likelihood of death increases with age in this population.

An altogether different model is needed, however, when studying survival with respect to a specific histologic type of tumour that has been treated by a potentially curative therapy. In order to focus on the tumour under study, distinction must be made between deaths due to this tumour and deaths due to unrelated causes. If reliable follow-up data is available, one can consider patients that died of other causes to be withdrawn alive at the time of death (Cutler & Axtell, 1969). Since risk of death from the tumour under study may eventually decline, as ascending hazard function would not give a good fit to observed survival (Figure 1). Furthermore, those patients that are cured are not at risk of death from their tumour, and thus a successful model must allow for a risk-free portion (or cured fraction) within the population.

This reasoning suggests that a model of tumour-specific survival can be constructed by combining a cured fraction with a distribution of time-to-death among uncured patients. One alternative is to approximate specific biologic pathways within the malignant process, using selected mathematical functions. Though this approach has become popular with the advent of sophisticated computer technology, it may demand numerous simplifying assumptions and the resulting conclusions, though sometimes useful, may apply only to the malignancy for which the model was derived (Birkhead, 1985; Gregory *et al.*, 1991). A more direct approach, and one with a long history, is the lognormal survival model. Originally present by Boag in 1949, this model used the lognormal function to approximate the distribution of time to death from tumour (Boag, 1949). The lognormal function has been shown to provide a good fit to clinical data for a variety of cancers, including those of breast, cervix, eye, and head and neck (Mould & Boag, 1975; Mould *et al.*, 1976; Rutqvist *et al.*, 1984; Gamel *et al.*, 1990). Because this model provides estimates of essential survival parameters (i.e., cured fraction [C], mean log survival time [M] and standard deviation survival time [S]), it enjoys an obvious advantage over models that are non-parametric with respect to time, such as the Cox regression (Cox, 1972).

Despite this advantage, the original model of Boag suffers a major deficiency: it does not allow for variations in cured fraction and mean log survival time among members of the study population. Thus to examine the impact of patient age or tumour stage, for example, one must subdivide the population into multiple subgroups and derive separate estimate of C and M for each group. Subdividing patients in this fashion reduces statistical power, especially if an attempt is made to determine simultaneously the impacts of multiple prognostic covariates.

Recently, a refinement of the original Boag model has been developed that allows C and M to be expressed as linear regressions on prognostic covariates (Gamel *et al.*, 1990), As a result of this refinement, cured fraction and mean log survival time can be allowed to vary from patient to patient, depending on such factors as age and sex of the patient, and stage and histologic characteristics of the tumour.

We will report on the results from this method when applied to data from 2,073 cases of squamous cell carcinoma of the head and neck.

### Methods

### *Patients studied*

Since 1963 it has been the policy of the Department of Otorhinolaryngology at the University of Liverpool to store in a prospective manner the data on all patients with head and neck tumours. This data base now includes 3,285 cases of histologically proven squamous carcinoma of the head and neck. Updating is performed at every patient episode, and cause of death is determined from personal records or from the Mersey Regional Cancer Registry. Each month a program is run to identify records with incomplete data.

Computerised records were reviewed for all patients seen at the Royal Liverpool Hospital between January 1963 and October 1991 with the diagnosis of squamous carcinoma of the head and neck. Of these, a total of 649 were omitted for one or more of the following reasons: last status was not known (37), degree of the histologic differentiation was not known (508), or node status was not known (104). For each

Correspondence: J.W. Gamel, Department of Ophthalmology & Visual Sciences, 301 E. Muhammad Ali Blvd., Louisville, KY, 40292, USA.
Received 4 July 1992; and in revised form 15 December 1992.

**Figure 1** The symbols represent actuarial tumour-related survival for the 2073 patients included in this analysis. The continuous line represents the multivariate lognormal survival model derived from this data set.

of the remaining 2,073 patients, the following covariates were known: sex of the patient, age of the patient at the time of initial treatment, degree of differentiation of the primary tumour, node status at the time of treatment, duration of followup, and status at last followup.

Host factors are shown in Table I and tumour factors are shown in Table II. The patient's performance status was classified by the Eastern Cooperative Oncology Group Method (A.J.C. Manual for staging of cancer, 1988). Both Primary tumour and neck node metastases (if any), were classified by the UICC method (UICC International Union Against Cancer, 1987). Where a resection was carried out, the specimen was examined macroscopically by a pathologist and then microscopically by a histologist and assigned an appropriate pT stage. Histologic grade was assessed according to the method generally used in the UK (Broders, 1926; Thomson, 1939) and assigned the category of well, moderately, or poorly differentiated.

In Table III, primary sites for the data base are given. Approximately one-third of the cases are carcinoma of the larnyx and a further third are carcinoma of the pharynx. One-fifth of cases has a carcinoma of the oral cavity. Tumours of the nose and sinuses, post-nasal space, ear, salivary glands, etc. form the remaining cases.

In Table IV the various forms of treatment are indicated. In the UK, the general policy of the Head and Neck Cancer Units is to treat by primary radiotherapy wherever possible, with the option of salvage surgery. In the present data base, 51% of all new patients were treated initially by radiotherapy with curative intent, but approximately one-third of these patients required salvage surgery. Of all patients, 29% were

**Table I** Patients: host factors

| | | |
|---|---|---|
| *Age* | Male | 61.8 years |
| | Female | 62.1 years |
| *Sex* | Male | 70% |
| | Female | 30% |
| | Total | 100% |
| *Performance status* | | |
| | 0 | 50% |
| | I | 16% |
| | II | 5% |
| | III | 1% |
| | IV | 2% |
| | Previous treatment elsewhere | 23% |
| | Not recorded | 3% |
| | Total | 100% |

**Table II** Patients: tumour factors

| | | |
|---|---|---|
| *Histology* | | |
| | Well differentiated | 31% |
| | Moderately well differentiated | 26% |
| | Poorly differentiated | 21% |
| | Not classified | 22% |
| | Total | 100% |
| *T Stage* | | |
| | $T_1$ | 28% |
| | $T_2$ | 16% |
| | $T_3$ | 21% |
| | $T_4$ | 11% |
| | Previously treated elsewhere | 23% |
| | Not classified | 1% |
| | Total | 100% |
| *N Stage* | | |
| | $N_0$ | 49% |
| | $N_1$ | 10% |
| | $N_{2a}$ | 5% |
| | $N_{2b}$ | 1% |
| | $N_{2c}$ | 4% |
| | $N_3$ | 6% |
| | Previously treated elsewhere | 23% |
| | Not classified | 2% |
| | Total | 100% |

**Table III** Sites

| | |
|---|---|
| Larnyx | 37% |
| Oral cavity | 21% |
| Hypopharynx | 18% |
| Oropharynx | 14% |
| Nose and sinuses | 4% |
| Post nasal space | 2% |
| Ear | 2% |
| Other | 2% |
| Total | 100% |

**Table IV** Treatment

| | |
|---|---|
| Radiotherapy | 51% |
| Surgery | 29% |
| No treatment | 17% |
| Other | 3% |
| Total | 100% |

treated initially by surgery with curative intent, while 17% of all patients were unsuitable for any form of treatment. A small proportion of patients required treatment with chemotherapy or other palliative measures.

Sex was coded as 1 for male and 2 for female. Age at the time of initial treatment was coded in years. Preliminary studies of degree of histologic differentiation revealed that the increment between well and moderately differentiated tumours had an impact similar to the increment between moderately and poorly differentiated tumours. Thus degree of differentiation was coded as 1 for well, 2 for moderately, and 3 for poorly differentiated. Node status was initially coded as 1 if no lymph nodes were found to be involved and as 2 if one or more nodes were found to be involved at the time of initial treatment. Further analysis using the univariate lognormal model demonstrated a substantially better fit to observed survival data (using the criterion of log likelihood function) if node status was coded simply as the number of positive nodes (range, 0–7), and thus this second untransformed value was adopted for survival analysis.

### *Data analysis*

The univariate and multivariate lognormal models have been described in detail elsewhere (Boag, 1949; Gamel *et al.*, 1990). For univariate analysis, each covariate was entered into both components of the lognormal model (i.e., cured fraction and mean log survival time). For multivariate analysis, all covariates were included in both components of the lognormal model. In order to demonstrate the relative prognostic value of all covariates, no step-down procedure was performed.

To determine the interaction of covariates, standard linear regression analysis was used. In order to assess the possible interaction of the studied covariates with therapeutic decisions, cross-correlation with type of therapy was also examined. For this analysis, surgery was coded as 2 if definitive resection was attempted and as 1 if there was no surgical intervention other than biopsy or palliative procedures. Radiation was coded as 1 if no radiation was given and as 2 if therapeutic (excluding palliative) radiation was performed.

### Results

Of the 2,073 cases included in survival analysis, 928 were coded as dead of their tumour at last followup, 500 were coded as dead of other causes, and 645 were coded as withdrawn alive. For those dead of their tumour, range of followup was 1 day to 13.0 years, with a median of 0.85 years, a mean of 1.30 years, and a standard deviation of 1.58 years. For those dead of other causes, the corresponding values were 4 days to 18.8 years, 2.159 years, 3.47 years, and 3.68 years respectively. For those withdrawn alive, the corresponding values were 1 day to 24.0 years, 5.64 years, 6.154 years, and 4.51 years respectively.

The results of univariate and multivariate lognormal analysis are shown in Table V. Cross-correlation among covariates is shown in Table VI. The clinical impact of covariates is demonstrated in Table VII.

### Discussion

Norris found that the most important predictor of survival in laryngeal cancer was the presence or absence of lymph node metastases (Norris, 1963). Stell noted that neck node status completely overshadows other prognostic factors in laryngeal carcinoma and that patients with nodal involvement were likely to have high T stage and poorly differentiated tumours (Stell, 1990*b*). Furthermore, he found that pathological N stage and the presence or absence of capsular rupture were of paramount prognostic importance.

Given the well-established predictive value of nodal status for patients with squamous carcinoma of the head and neck

**Table VI** Cross-correlation among covariates for 2,073 patients with squamous cell carcinoma of the head and neck

| *Covariate* | *Correlation Coefficient (r)*[a] *Age* | *Sex* | *Hist. grade* | *Node status* |
|---|---|---|---|---|
| Hist. Grade | −0.03 | +0.05 | | |
| Node Status | −0.08 | −0.05 | +0.12 | |
| Surgery | −0.12 | +0.02 | +0.01 | −0.01 |
| Radiation | −0.04 | −0.02 | −0.02 | −0.28 |

[a]For $|r|>0.04$, $P<0.05$; for $|r|>0.06$, $P<0.005$; for $|r|>0.075, P<0.0001$.

**Table V** Maximum-likelihood estimates of coefficients of lognormal model

| *Covariate (Parameter)* | *Univariate analysis* *Constant* | *Covar.* | *SE* | *t-value*[a] | *Multivariate analysis* *Constant* | *Covar.* | *SE* | *t-value*[a] |
|---|---|---|---|---|---|---|---|---|
| (Cured fraction) | | | | | | | | |
| Sex | −0.112 | −0.231 | 0.139 | 1.663 | 1.903 | −0.336 | 0.154 | 2.175 |
| Age | 0.064 | −0.007 | 0.006 | 1.287 | – | −0.012 | 0.006 | 1.844 |
| Hist. Gr. | 0.398 | −0.454 | 0.087 | 5.215 | – | −0.434 | 0.097 | 4.463 |
| Node St. | −0.067 | −0.510 | 0.080 | 6.332 | – | −0.565 | 0.104 | 5.411 |
| (Mean log survival time) | | | | | | | | |
| Sex | 0.241 | −0.086 | 0.122 | 0.707 | 2.378 | −0.092 | 0.114 | 0.811 |
| Age | 1.830 | −0.027 | 0.005 | 5.611 | – | −0.032 | 0.005 | 6.740 |
| Hist. Gr. | 0.101 | 0.022 | 0.072 | 0.303 | – | 0.072 | 0.067 | 1.073 |
| Node St. | 0.389 | −0.175 | 0.025 | 7.107 | – | −0.182 | 0.024 | 7.554 |

[a]$df=\infty$; $t=1.692$ and $t=2.81$ correspond to $P$-values of 0.05 and 0.005 respectively.

**Table VII** Clinical impact of covariates estimated by multivariate lognormal model[a]

| *Sex* | *Age (Yrs)* | *Hist. grade* | *Number positive nodes* | *Cured fraction* | *Median survival time (yrs)* |
|---|---|---|---|---|---|
| Female | 30 | Well | 0 | 0.685 | 4.09 |
| Male | 30 | Well | 0 | 0.608 | –[b] |
| Female | 60 | Well | 0 | –[b] | 1.58 |
| Female | 30 | Poorly | 0 | 0.477 | –[b] |
| Female | 30 | Well | 1 | 0.552 | 3.41 |
| Female | 30 | Well | 3 | 0.285 | 2.37 |

[a]First row represents baseline values; each other row has only one covariate changed from baseline.
[b]In the multivariate lognormal model, the changed covariate was not statistically significant in its association with this parameter.

(SCHN), it is not surprising to find that this variable is highly associated with both cured fraction and survival time. The biologic principles underlying this association are apparent: likelihood of cure is significantly diminished by the dissemination of tumour to adjacent or regional lymph nodes, while the dissemination of tumour cells to lymph nodes by the time of initial evaluation can be expected to shorten the time between initial evaluation and death from tumour.

As can be seen in Table V, histologic type and age are also important predictors for SCHN. Unlike nodal status, however, these two covariates are asymmetric predictors– histologic type is significantly associated only with cured fraction, while age is significantly associated only with median survival time.

Kleinsasser in 1961 noted that undifferentiated carcinoma of the larnyx has a poor prognosis and this association was attributed to the fact that these tumours metastasise early. Stell, however, (Stell, 1990*b*) found histological differentiation to be nonsignificant regarding survival after allowing for confounding variables. Our finding that histologic type is associated with cured fraction rather than survival time may explain this apparent contradiction.

The existence of highly asymmetric predictors such as age and histologic type suggest an important conclusion–that those mechanisms which govern the likelihood of metastasis are distinct from those that govern survival time for uncured patients. Specifically, poorly differentiated cells within SCHN are apparently more likely to metastasise than well differentiated cells, but these metastatic foci do not seem to grow more quickly than their better differentiated counterparts. On the other hand, old age has a highly significant interaction with survival time but not with cured fraction, suggesting the possibility that rate of metastatic proliferation, rather than the occurrence of metastasis, increases substantially with increasing age at diagnosis. A second possibility is age-associated delay in diagnosis; this association appears unlikely, however, since the multivariate model contains a covariate (node status) that characterises tumour stage at diagnosis and thus should control for delay in diagnosis. A third possibility is that older patients succumb at a relatively smaller tumour burden than younger patients. It should be noted that age also demonstrated a strong and asymmetric association with survival time for patients with melanoma of the skin and of the eye (Gamel *et al.*, in preparation *a,b*).

For many years it has been assumed that prognosis from SCHN is better in younger than older patients (Lauerma, 1967). This assumption has been borne out by careful statistical investigations (Huygen *et al.*, 1980; Katz, 1983). On the other hand, with use of the Cox regression model Stell found that age was not a significant predictor of survival when allowance was made for those patients that were untreated or died of intercurrent disease or of a second primary tumour (Stell, 1990*a*). This disparity may result from a difference in statistical methods.

Before attributing biologic significance to these observations, however, it is important to consider the interactions among covariates shown in Table VI. These interactions imply that patients with nodal involvement are less likely to have radiation therapy and more likely to have poorly differentiated tumours than those without nodal involvement. Old patients are also less likely than young patients to have surgery and less likely to have nodal involvement. Some of these associations are of marginal biologic significance–e.g., only 0.64% of the variation in age can be explained by node status.

Nevertheless, because of the large number of patients included in this study, there may be sufficient statistical significance to impact patient survival. Thus the interaction of covariates with therapy and with each other can significantly affect their prognostic value within the lognormal model.

Because of these limitations, are because of the limitations that arise from the complexities of multivariate survival analysis itself, no firm conclusions can be drawn from the results shown in Table V. Nevertheless, these findings raise important biologic questions that warrant further investigation, both in the clinic and in the laboratory.

This work was supported by a Veterans Administration Merit Review Research Grant Project 001, the Kentucky Lions Eye Foundation, and an unrestricted grant from Research to Prevent Blindness, Inc. (New York, New York).

The opinions or assertions contained herein are the private views of the authors and are not to be construed as official or as reflecting the views of the US Government.

### References

(1987). UICC International Union Against Cancer. TNM classification of malignant tumours, 4th edition. Springer Verlag: Heidelberg.

(1988). A.J.C. Manual for staging of cancer, 3rd edition, J.B. Lippincott: New York.

BIRKHEAD, B.G. (1985). Mathematical model of remission duration in acute myelogenous leukemia. *Cancer Treat. Rep.*, **69**, 595–601.

BOAG, J.W. (1949). Maximum likelihood estimates of the proportion of patients cured by cancer therapy. *J. Roy. Stat. Soc. [B]*, **22**, 15–44.

BRODERS, A.C. (1926). Carcinoma grading and practical application. *Arch. Path. (Chicago)*, **2**, 576–581.

COOK, P., DOLL, R. & FELLINGHAM, S.A. (1969). A mathematical model for the age distribution of cancer in man. *Int. J. Cancer*, **4**, 93–112.

COX, D.R. (1972). Regression models and life tables (with discussion). *J. Roy. Stat. Soc. [B]*, **34**, 187–220.

CUTLER, S.J. & AXTELL, L.M. (1969). Adjustment of long-term survival rates for deaths due to intercurrent disease. *J. Chronic Dis.*, **22**, 485–491.

GAMEL, J.W., MCLEAN, I.W. & MCCURDY, J.B. Biologic distinctions between cure and time to death in 2892 patients with intraocular melanoma. *Cancer* (in press) [*a*].

GAMEL, J.W., MCLEAN, I.W. & ROSENBERG, S.H. (1990). Proportion cured and mean log survival time as functions of tumour size. *Stat. Med.*, **9**, 999–1006.

GAMEL, J.W., SEIGLER, H.F., GEORGE, S.L. & STANLEY, W. Parametric and non-parametric analysis of survival among 1888 patients with skin melanoma. In preparation [*b*].

GREGORY, W.M., RICHARDS, M.A., MAURICE, L.S. & SOUHAMI, R.L. (1991). A mathematical model relating response durations to amount of subclinical resistant disease. *Cancer Res.*, **51**, 1210–1216.

HUYGEN, P.L.M., VAN DER BROEK, P. & KAZEM. I. (1980). Age and mortality in laryngeal cancer. *Clin. Otolaryngol.*, **5**, 129–137.

KATZ, A.E. (1983). Immunobiologic staging of patients with carcinoma of the head and neck. *The Laryngoscope*, **93**, 445–463.

KLEINSASSER, O. (1961). Ein larynxmikroskop zur fruehdiagnose und differential diagnose von kreben in kehlkopf, rachen und mundhohle. *Laryng. Rhinol. Otol.*, **40**, 276–279.

LAUERMA, S. (1967). Treatment of laryngeal cancer. A study of 638 cases. *Acta Otolaryngol.* (suppl), **225**, 1–67.

MOULD, R.F. & BOAG, J.W. (1975). A test of several parametric statistical models for estimating success rate in the treatment of carcinoma cervix uteri. *Br. J. Cancer*, **32**, 529–550.

MOULD, R.F., HEARNDEN, T., PALMER, M. & WHITE, G.C. (1976). Distribution of survival times of 12,000 head and neck cancer patients who died with their disease. *Br. J. Cancer*, **34**, 180–190.

NORRIS, C.M. (1963). Problems in classification and staging of cancer of the larynx. *Ann. Otol.*, **72**, 93–96.

PIKE, M.C. (1966). A method of analysis of a certain class of experiments in carcinogenesis. *Biometrics*, **22**, 142–161.

RUTQVIST, L.E., WALLGREN, A. & NILSSON, B. (1984). Is breast cancer a curable disease? A study of 14731 women with breast cancer from the cancer registry of Norway. *Cancer*, **53**, 1793–1800.

STELL, P.M. (1990*a*). Prognosis in laryngeal carcinoma: host factors. *Clin. Otolaryngol.*, **15**, 111–119.

STELL, P.M. (1990*b*). Prognosis in laryngeal carcinoma: tumour factors. *Clin. Otolaryngol.*, **15**, 69–81.

THOMSON, S.C. (1939). The history of cancer of the larynx. *J. Larnygol.*, **54**, 64–87.